# Stunting and Its Determinants among Children Aged 6–59 Months in Northern Ethiopia: A Cross-Sectional Study

**DOI:** 10.1155/2018/1078480

**Published:** 2018-06-25

**Authors:** Shiferaw Abeway, Bereket Gebremichael, Rajalakshmi Murugan, Masresha Assefa, Yohannes Mehretie Adinew

**Affiliations:** ^1^Amhara Regional Health Bureau, Bahardar, Ethiopia; ^2^Department of Nursing and Midwifery, Allied School of Health Science, College of Health Science, Addis Ababa University, Addis Ababa, Ethiopia; ^3^College of Health Sciences and Medicine, Wolaita Sodo University, Sodo, Ethiopia

## Abstract

**Background:**

Stunting reflects chronic undernutrition during the most critical periods of growth and development in early life. The study was aimed at assessing the magnitude of stunting and associated factors among children aged 6–59 month in central Ethiopia.

**Methods:**

A community-based cross-sectional study was conducted among 410 children aged between 6 and 59 months. Systematic random sampling technique was employed to select study participants. Interviewer-administered structured questionnaire was used to collect data. The data were entered using EPI INFO version 3.5.1, and analysis was done by SPSS version 21 and ENA, 2007 software for anthropometric calculation. World Health Organization standard 2006 was used to analyse anthropometric data. Bivariate and multivariable logistic regression analyses were also carried out to identify predictors of stunting. Statistical significance was declared at *p* < 0.05 and 95% CI.

**Results:**

Overall magnitude of stunting was 52.4 (95% CI: 47.6–57.2). Being female (AOR: 2.8, 95% CI: 1.503–5.099), belonging to age group of 25–59 months (AOR: 4, 95% CI: 1.881–8.424) and birth weight of <2.5 kg (AOR: 5, 95% CI: 1.450–17.309), mothers' lack of ANC visits (AOR: 3.2 95% CI: 1.40–7.10), and mistimed complementary feeding initiation (AOR: 2.4, 95% CI: 1.266–4.606) were positively associated with child stunting, whereas educational status of the mother (AOR: 0.01, 95% CI: 0.001–0.063) showed negative association.

**Conclusion:**

Stunting was a highly prevalent problem in the study area. Low weight at birth, female sex, older age, mistimed initiation of complimentary feeding, and mothers' lack of ANC visit were found to have significant relation with children's chronic malnutrition. Thus, interventions shall effectively address those factors to alleviate the problem.

## 1. Background

Malnutrition results from unbalanced diet that does not contain all the necessary nutrients and/or inadequate or excessive consumption of nutrients. It can also result from diseases that interfere with the body's ability to use the nutrients consumed [1] and thus malnourished children have lowered resistance to infection [2]. Stunting reflects chronic undernutrition during the most critical periods of growth and development in early life. Children whose height-for-age *z*-score is below minus two standard deviations (−2 SD) from the median of the World Health Organization (WHO) reference population are considered short for their age (stunted), or chronically malnourished. Children who are below minus three standard deviations (−3 SD) are considered severely stunted [3].

Although problems related to poor nutrition affect the entire population, children are more vulnerable because of their unique physiology and socioeconomic characteristics. Adequate nutrition is critical to children's growth and development. The period from birth to age two is especially important for optimal physical, mental, and cognitive growth, health, and development. Unfortunately, this period is often marked by protein energy and micronutrient deficiencies that interfere with optimal growth [4].

Undernutrition affects children's physical growth and reduces their cognitive development, and physical work capacity. It is also responsible for at least 35% of deaths in under-five children globally [1]. Every hour of every day, 300 children die because of malnutrition but it's not recorded on death certificates, and as a result, it's not effectively addressed [5, 6]. Worldwide, 165 million children below five years of age are affected with undernutrition, of which 26% are stunted [7].

Progress in reducing childhood malnutrition in developing countries has been slow [8]. The larger burden for Africa is stunting, failure to grow in stature [5, 9] with 36% prevalence [10]; 40% in rural areas and 25% in urban [3, 10]. Ethiopia's success is also limited with an annual reduction of 1.3% over the past eleven years from 58% in 2000 to 44% in 2011 and 40% in 2014 [5]; huge gap in achieving the goal of stunting. Especially stunting level is above the national average in Amhara region 46% [3]. According to recent studies conducted in the region, the prevalence of child stunting was 14.3% in west Gojam [1], 31.1% in southeast Amhara region [11], and 51.1% in Lalibela [12]. Malnutrition is responsible for 57% of child deaths in Ethiopia, the highest rates of stunting in the world [13]. Therefore, identifying factors underlying stunting is crucial. Thus, this study was aimed to determine the magnitude of stunting and its predictors in under-five children.

## 2. Methods

### 2.1. Study Design and Area

A community-based cross-sectional study was conducted from February to March 2017. The study was conducted in Merhabete woreda (district), North Shewa zone, 180 km away from the capital Addis Ababa [14].

### 2.2. Sample Size Determination and Sampling Technique

Sample size was determined using single population proportion formula with assumption of 51.1% prevalence of stunting [12], 5% margin of error, 95% confidence level, and 10% possible nonresponse rate, making the final sample size 422 mother child pair.

The study population was all randomly selected children aged 6–59 months who were living with their mothers in selected kebeles (the lowest administrative unit) of Merhabeta woreda. From the total of 27 kebeles in the woreda, nine were selected by simple random sampling method. A sample frame of each selected kebeles (number of under-five children in each kebeles) was obtained from woreda health bureau. Samples were allocated to all kebeles proportional to the size or number of under-five children they had. List of participating households from the selected kebeles was obtained from health extension workers, and participants were enrolled by using systematic random sampling technique. Interval (*K* value) was determined for each kebele by dividing the total eligible children in the kebele to the sample proportion. The first household was selected by lottery method. In case there was more than one eligible child in one home, one child was arbitrarily selected. Children, who were seriously ill, had physical deformities of limbs and spines were excluded because of difficulty in height measurement.

### 2.3. Operational Definitions

#### 2.3.1. Stunting

Children who had low height-for-age at <−2 SD of the median value of the WHO international growth reference aged 6–59 months were selected.

### 2.4. Data Collection Tools and Procedure

Eight diploma nurses collected the data under supervision of two B.Sc nurses. Data were collected by using semistructured questioner adopted from UNICEF and similar studies [5]. The tool contains demographic, stunting (chronic malnutrition), socioeconomic, environmental, healthcare, and dietary factors among children aged 6–59 months.

#### 2.4.1. Height

Length measurement for children below 24 months was taken in lying down or recumbent position, and standing height was taken for children aged 24–59 months, and the measurement was taken to the nearest 0.1 cm using Short's Height Measuring Board [5]. Study participants were on barefoot while measuring their height.

#### 2.4.2. Age

Mothers' response and birth certificate or vaccination cards were used to record child's age. “Local-events” were used to help mothers remember.

### 2.5. Data Quality Control

Appropriate training and supervision were given to data collectors. Pretest was performed using 5% of the sample size in adjacent kebele not included in the survey. The questionnaire was translated to local language (Amharic) and back translated to English to check for internal consistency. Height scale was calibrated at the nearest 0.1 cm using Short's Height Measuring Board, and continuous checkup of measurement was performed for their reliability.

### 2.6. Data Processing and Analysis

After checking for completeness and consistencies, the data were entered using Epi Info and analyzed by SPSS version 21. Nutritional status of the child/height-for-age *z* score was computed for each sex based on the Emergency Nutritional Assessment (ENA) 2007 software for anthropometric calculator and WHO standard 2006. Descriptive statistics were done. All variables with association (*p* < 0.2) in the binary logistic regression analysis were interred into the final multivariable regression model to identify their independent effect. Statistical significance was declared at *P* < 0.05.

## 3. Results

### 3.1. Demographic and Socioeconomic Characteristics

Out of eligible 422 children with their mother, a total of 410 actually participated in the study making the response rate 97.2%. More than half, 229 (55.9%), of the children were male and 167 (73.2%) of them were in the age group of 25–59 months. Among respondents, 399 (97.3%) and 404 (98.5%) were Amhara in ethnic group and orthodox religion followers, respectively. Nearly two-thirds, 259 (63.2%), of mothers had no formal education (Table 1).

### 3.2. Health-Care and Environmental Characteristics

Half, 211 (51.5%), of the children had normal birth weight (2.5–4.0 kg) and 15 (3.7%) were >4.0 kg, while 133 (32.4%) of the total children were immunized. Among the children, 166 (40.5%) had got diarrhoea during the last two weeks prior to data collection and 168 (41%) children were affected by recurrent diarrhoea. Almost half of the mothers, 186 (45.4%), had no antenatal care visits for their last pregnancy (Table 2).

### 3.3. Dietary Characteristics

Breastfeeding was almost universal 401 (97.8%) in the study area. Majority (82.7%) of children started breastfeeding within the first one hour of birth. Children who received colostrum were 165 (40.2%), whereas 121 (29.5%) children received prelacteal feed. Majority, 233 (58.8%), of children started complementary feeding at age of 6 months (Table 3).

### 3.4. Prevalence of Stunting among Children Aged 6–59 Months

Prevalence of stunting was found to be 215 (52.4%) (95% CI: 47.6–57.25). Of this, 182 (84.7%) were in the age group of 25–59 month. From the total of stunted children, 74 (18%) were severely stunted and 141 (34.4%) were moderately stunted.

### 3.5. Factors Associated with Stunting among Children Aged 6–59 Months

In multivariable logistic regression analysis, birth weight, sex, and age of the child, educational status, and ANC visits of mother and age at which the child started complementary food were identified as factors associated with stunting among children aged 6–59 months in the study area.

Children within age group 25–59 months were 4 times (AOR: 4; 95% CI: 1.88–8.42) more likely to be stunted compared to children in age group 6–24 months, and children whose mothers attended higher education had reduced risk of being stunted by 90% compared to mother who cannot read and write (AOR: 0.01; 95% CI: 0.01–0.06). Female children were 2.8 times (AOR: 2.8; 95% CI: 1.50–5.10) more likely to be stunted than their male counterparts. Children who did not have timely start of complementary feeding were 2.4 times (AOR: 2.4; 95% CI: 1.27–4.61) more likely to be stunted than children who started at 6 months of age (Table 4).

## 4. Discussion

The study assessed prevalence of stunting and associated factors among children of 6–59 months. Prevalence of stunting in the study area was 52.4%. The finding was almost comparable with studies conducted in Amhara region (Lalibela town) (51.1%) [12], Sidama zone (50.3%) [15], and Oromia Regional State (47.6%) [16]. The prevalence was relatively higher than the national figure (38%) [10] and other parts of the country, 26.6% in southern region [1] and 24.9% in Northwest Ethiopia [6]. However, it was lower than the finding from Southeast Amhara region (60.6%) [11].

Sex of a child showed significant association with stunting. Female children had higher odds of stunting than male. This was congruent with the study from rural Somalia, where females were more stunted than their male counterparts [17]. Another study from Pakistan also found increased stunting among the female [18]. This variation might be due to unmeasured factors on care-giving behaviours of mothers because of preference. In country like Ethiopia, parents are stricter with their daughters than their sons and often parents give more meal freedom to male children than females [17].

Children's age was the other determinant of stunting, as evidenced by higher odds of stunting among the older age ones. Compared with those children aged 6–24 months, children within age group 24–59 months were 4 times more like to be stunted. This result is consistent with result of study from Hawasa, where children who were aged above 24 months were more stunted than those below 24 months [1]. This could be due to the fact that stunting is a chronic malnutrition and is commonly manifested after long-term nutritional deprivation.

Child birth weight has a direct relationship with stunting. Small birth weight children were more likely to be stunted than normal birth weight children. Similar findings were obtained in the East Wolega zone [19]. The study conducted in Nepal [20] and Sri Lanka [21] also showed disproportionately higher prevalence of stunting among low birth weight children. This could be related to mother's health and nutritional status before and during pregnancy which determine the size of the child during intrauterine period and also those small-sized children were likely be frequently exposed to infections which lead to malabsorption of nutrients in their body.

Starting complimentary food at the right time has a positive effect on child development. Children who did not have timely complementary feeding were more likely to be stunted than children who had started complementary feeding at 6 months. Similar findings were obtained in West Gojam [7], Khartoum [22], and Belgaum [23]. This could be explained by the fact that breastfeeding is enough to provide child's nutritional demand during the first six months life. On the contrast, as the duration of exclusive breastfeeding extends from the recommended duration, the risk of chronic malnutrition increases because of low intake of energy rich supplementary food [24, 25].

Maternal education appeared to strongly affect childhood stunting. Children born to mothers with higher education had reduced risk of stunting compared to those born to illiterate mothers. This finding is in agreement with the study conducted in the Sidama zone that reported higher risk of stunting among children of uneducated mothers [15]. Studies from Brazil [26] and Zambia [27] had also supported this finding. Educated women are more likely to discern and practice appropriate child nutrition, hygiene, and health care which can greatly improve the nutritional status of their children.

Mothers' ANC visit also showed strong association with child stunting. Children whose mothers had no antenatal follow-up were more likely to be stunted than children whose mothers had four and above ANC visits. The finding is supported by the study conducted in Southeast Amhara region where children of mothers who had no antenatal care were more stunted than those who had two to three ANC visit [11]. A study from Zambia also found reduced risk of stunting among children whose mothers had attended antenatal clinics compared to those whose mothers had no ANC [28]. Nutritional intervention and maternal advice during pregnancy had a great impact on child growth and multiple causes of stunting. This includes prevention and control of prenatal infection and subclinical condition that restrict growth of children and stimulate early child development. In addition, mothers get advises from health-care providers on breast feeding, child nutrition, and infection prevention during ANC follow-up which could help them raise their children better [29].

## 5. Limitations

Birth weight data were collected by interviewing the mothers or caregivers, and it might be subjected to recall bias.

## 6. Conclusion

Stunting was prevalent among Merhabete woreda children. Low weight at birth, female sex, and older age, mistimed initiation of complimentary feeding, mothers' low educational status, and lack of ANC visit were found to have significant relation with child chronic malnutrition. Thus, interventions shall effectively address these factors to alleviate the problem stunting.

## Figures and Tables

**Table 1:** Demographics and socioeconomic characteristics of children aged 6 to 59 months in Merhabete woreda, Ethiopia, 2017 (*n*=410).

Variable, *n*=410	Category	Frequency (N)	Percent (%)
Age of child	6 to 24 months	107	26.1
	25 to 59 months	303	73.9

Sex of child	Male	228	55.9
	Female	182	44.1

Birth order	First child	149	36.4
	2-3	27	6.6
	4-5	26	6.3
	4=>6	208	50.7

Birth interval	1st child	150	36.6
	<24 months	227	55.4
	>24 months	33	8

Age of mother	Less than 20	14	3.4
	21 to 35	360	87.8
	Greater than 36	36	8.8

Marital status	Married	373	91.0
	Single	3	0.7
	Divorced	23	5.6
	Widowed	11	2.7

Ethnicity	Amhara	399	97.3
	Oromo	11	2.7

Religion	Orthodox	404	98.5
	Protestant	6	1.5

Occupation of mother	Housewife	370	90.2
	Government employee	27	6.6
	NGO	3	0.7
	Merchant	6	1.5
	Self-employee	4	1.0

Educational status of mother	No formal education	259	63.2
	Primary school	116	28.3
	Secondary school	23	5.6
	Above 12	12	2.9

Educational status of father	No formal education	143	34.9
	Primary school	118	28.8
	Secondary school	110	26.8
	Above 12	39	9.5

Family monthly income	<1000 ETB	127	30.9
	1000–2000 ETB	145	35.4

**Table 2:** Environmental and health-care characteristics of children aged 5 to 59 months in Merhabete woreda, Ethiopia, 2017 (*n*=410).

Variable, *n*=410	Category	Frequency (N)	Percent (%)
ANC follow-up	No	186	45.4
	1 times	82	20
	2-3 times	14	3.4
	4 times	128	31.2

Place of delivery	Health center	40	9.8
	Hospital	330	80.5
	Privet health center	18	4.4
	Home	22	5.3

PNC follow-up	Yes	134	32.7
	No	276	67.3

Birth weight	<2.5 kg	162	39.5
	2.5–4 kg	211	51.5
	>4 kg	15	3.7
	Unknown	22	5.4

Immunization status	Yes	133	32.4
	No	277	67.6

Category of vaccination	Fully vaccinated	93	22.7
	Currently on vaccination	40	9.8
	Not fully vaccinated	277	67.5

Had recurrent diarrhoea in the past 2 weeks	Yes	168	41
	No	242	59

Diarrhoea within 2 weeks	Yes	166	40.5
	No	244	59.5

Main source of water	Private tap/stand pipe	138	33.7
	Public tap/stand pipe	265	64.6
	Hand pump water	5	1.2
	Protected dwelling	2	0.5

Toilet facility availability	Yes	331	80.7
	No	79	19.3

**Table 3:** Dietary characteristics of children among 6 to 59 months in Merhabete woreda, Ethiopia, 2017, (*n*=410).

Variable, *n*=410	Category	Frequency (N)	Percent (%)
Ever breastfed child	Yes	401	97.8
	No	9	2.2

Time for initiation of BF	Within 1 hr	339	82.7
	Within 24 hrs	57	13.9
	>24 hrs	14	3.4

Child fed colostrum	Yes	165	40.2
	No	245	59.8

Child received prelacteal feed	Yes	121	29.5
	No	289	70.5

Age complementary food started	At 6 months	233	56.8
	Not timely initiation of BF	177	43.2

Type of food offered	Bread	14	3.4
	Enjera	257	62.7
	Porridge	118	28.8
	Other	21	5.1

Duration of breastfeeding	Less than 12 months	94	22.9
	12 to 24 months	278	67.8
	Greater than 24 months	38	9.3

**Table 4:** Bivariate and multivariable logistic regression analysis of factors associated with stunting among children aged 6–59 months in Merhabete woreda, Ethiopia, 2017 (*n*=410).

Variable	Stunted	Nonstunted	COR (95% CI)	AOR (95% CI)	*P* value
*Sex*					
Male	105 (48.8%)	123 (63.1%)	1	1	
Female	110 (51.2%)	72 (36.9%)	1.79 (1.20–2.65)	**2.8 (1.50–5.09**)	**0.00**

*Birth order*					
First child	54 (25.1%)	95 (48.7%)	1	1	
2-3	15 (7%)	12 (6.2)	2.2 (0.96–5.04)	0.5 (0.20–1.08)	0.07
4-5	15 (7%)	11 (5.6)	2.4 (1.02–5.59)	3.1 (0.98–9.92)	0.05
>6	131 (60.9%)	77 (39.5%)	3 (1.99–4.63)	1 (0.31–3.48)	0.94

*Birth interval*					
First child	70 (32.6%)	80 (41%)	1.5 (0.70–3.33)	1.7 (0.45–6.37)	0.43
<24 months	133 (61.9%)	94 (48.2%)	2.5 (1.16–5.27)	1 (0.29–3.17)	0.96
>24 months	12 (9.8%)	21 (10%)	1	1	

*Education of mother*					
No formal	164 (76.3%)	95 (48.7%)	1	1	
Primary	46 (21.4%)	70 (35.9%)	0.4 (0.24–0.59)	**0.2 (0.10–0.42)**	**0.00**
Secondary	4 (19.1%)	19 (9.7%)	0.1 (0.04–0.36)	**0.1 (0.02–0.44)**	**0.00**
Above 12	1 (15.8%)	11 (0.5%)	0.05 (0.00–0.41)	**0.01 (0.00–0.06)**	**0.00**
*ANC*					
No	132 (61.4%)	54 (27.7%)	6.5 (3.93–10.72)	**3.2 (1.40–7.10)**	**0.00**
1 time	44 (20.5%)	38 (19.5%)	3.1 (1.71–5.51)	2.4 (1.00–5.74)	0.05
2-3 times	4 (1.9%)	10 (5.1%)	1.1 (0.313–0.3.611)	1.2 (0.24–5.39)	0.85
>4 times	35 (16.3%)	93 (477%)	1	1	

*PNC*					
Yes	48 (22.3%)	86 (44.1%)	1	1	
No	167 (77.7%)	109 (55.9%)	2.7 (1.79–4.21)	0.6 (0.02–11.93)	0.71

*Birth weight*					
<2.5	163 (75.7%)	48 (24.6%)	4 (1.65–10.01)	**5 (1.45–17.30)**	**0.01**
2.5–4	48 (22.4%)	136 (69.8%)	0.4 (0.14–0.91)	0.4 (0.11–1.46)	0.17
>4	4 (1.9%)	11 (5.6%)	1	1	

*Immunization*					
Yes	47 (21.9%)	85 (43.6%)	1	1	
No	168 (78.1%)	110 (56.4%)	2.8 (1.84–4.33)	1.7 (0.08–20.37)	0.73

*Recurrent episode of diarrhoea*					
Yes	112 (52.1%)	56 (28.7%)	2.7 (1.79–4.06)	1.7 (0.71–4.18)	0.22
No	103 (47.9%)	139 (71.3%)	1	1	

*Diarrhoea*					
Yes	110 (51.2%)	56 (28.7%)	2.6 (1.72–3.91)	1.1 (0.45–2.60)	0.85
No	105 (48.8%)	139 (71.3%)	1	1	

*Colostrum feed*					
Yes	65 (30.2%)	100 (51.3)	1	**1**	
No	150 (69.8%)	95 (48.7%)	2.4 (1.62–3.64)	0.8 (0.41–1.46)	0.43

*Method of feeding*					
Spoon	22 (10.2%)	19 (9.7%)	1.5 (0.53–4.45)	0.4 (0.06–2.13)	0.26
Cup	99 (46%)	109 (55.9%)	0.6 (0.48–0.99)	0.5 (0.10–2.11)	0.32
Hand	85 (39.5%)	55 (28.2%)	2.0 (0.81–5.21)	0.6 (0.13–2.94)	0.54
Bottle	9 (4.2%0	12 (6.2%)	1	1	

*Age of child*					
6 to 24 months	33 (15.3%)	74 (37.9%)	1	**1**	
25 to 59 months	182 (84.7%)	121 (962.1%)	3.4 (2.10–5.39)	**4 (1.88–8.42)**	**0.00**

*Duration of BF*					
<12 months	36 (16.7%)	58 (29.7(%)	0.5 (0.23–0.97)	1.1 (0.34–3.67)	0.85
12 to 24 months	158 (73.5%)	120 (61.5%)	1.1 (0.53–2.10)	1.4 (0.51–3.87)	0.51
>24 month	21 (9.8%)	17 (8.7%)	1	1	

*Complementary food started*					
At 6 months	81 (37.7%)	152 (77.9%)	1	1	
Before and after 6 months	134 (62.3%)	43 (22.1%)	5.6 (3.77–9.05)	**2.4 (1.26–4.66)**	**0.00**

## Data Availability

Data supporting the findings of this study are available and can be accessed with reasonable request.

## Abbreviations

ANC: Antenatal care
UNICEF: United Nations Children's Fund
WHO: World Health Organization.
